# NMR structures and mutational analysis of the two peptides constituting the bacteriocin plantaricin S

**DOI:** 10.1038/s41598-019-38518-6

**Published:** 2019-02-20

**Authors:** Bie Ekblad, Per Eugen Kristiansen

**Affiliations:** Department of Biosciences, University of Oslo, PO Box 1066, Blindern, NO-, 0316 Oslo, Norway

## Abstract

The structure of the individual peptides of the two-peptide bacteriocin plantaricin S, an antimicrobial peptide produced by a *Lactobacillus plantarum* strain, has been determined in DPC micelles. The two peptides of plantaricin S, Pls-α and Pls-β, form an α-helix from and including residue 8 to 24 with a less structured region around residue 16-19 and an amphiphilic α-helix from and including residue 7 to 23, respectively. Activity assays on single amino acid-substituted GxxxG and GxxxG-like motifs show that substituting the Ser and Gly residues in the G_9_xxxG_13_ motif in Pls-α and the S_17_xxxG_21_ motif in Pls-β reduced or drastically reduced the antimicrobial activity. The two-peptide bacteriocin muricidin contains GxxxG-like motifs at similar positions and displays 40-50% amino acid identity with plantaricin S. Activity assays of combinations of the peptides that constitute the bacteriocins plantaricin S and muricidin show that some combinations are highly active. Furthermore, sequence alignments show that the motifs important for plantaricin S activity align with identical motifs in muricidin. Based on sequence comparison and activity assays, a membrane-inserted model of plantaricin S in which the two peptides are oriented antiparallel relative to each other and where the GxxxG and GxxxG-like motifs important for activity come close in space, is proposed.

## Introduction

In view of the dramatic increase in antibiotic-resistant bacteria, the development of new antimicrobial drugs and food preservatives that kill and/or prevent growth of pathogenic bacteria is of increasing importance^1,2^. Ribosomally synthesized antimicrobial peptides (AMPs) are produced by organisms ranging from bacteria to plants and mammals^3–6^. Bacterially produced AMPs, generally referred to as (peptide-) bacteriocins, are very potent, being active at pico- to nanomolar concentrations, and have been proposed as an alternative to commonly used antibiotics^5^. Bacteriocins produced by lactic acid bacteria (LAB) have received special attention due to LABs being generally recognized as safe, as they are present in the human diet and intestinal microbiota. The LAB bacteriocins nisin and pediocin PA-1 are presently used as bio-preservatives in food, the former being approved in over 40 countries for use as a food additive^5^. Bacteriocins have been grouped into two major classes: class I, the lanthionine-containing bacteriocins such as nisin, and class II, the non-lanthionine-containing bacteriocins such as pediocin PA-1. Class II is further subdivided into pediocin-like bacteriocins (class IIa), two-peptide bacteriocins (class IIb), cyclic bacteriocins (class IIc) and the non-pediocin-like one-peptide bacteriocins (class IId)^7^. The two-peptide bacteriocins are unique in that they, as the name suggests, require two different peptides in equal amounts to exert optimal antimicrobial activity, whereas the individual peptides have no or very low antimicrobial activity^7,8^.

*Lactobacillus plantarum* strains produce at least four different class IIb bacteriocins. While the structure and interactions of plantaricin EF and to some extent plantaricin JK are well investigated, a lot less is known about plantaricin NC8 and plantaricin S. Plantaricin S consists of the 27 residue Pls-α peptide and the 26 residue Pls-β peptide and is produced by *Lab. plantarum* strains involved in olive fermentation^9–11^. It is mainly active (in nanomolar range) against closely related strains of the producer as well as natural competitors found in olive fermentation and some strains of the olive spoilage bacterium *Enterococcus faecalis*^9,12^. Genetic characterization of the genes involved in bacteriocin production and secretion has been partially elucidated in the strain *Lab. plantarum* LPCO10^13^.

Recent results indicate that many, if not all, of the LAB bacteriocins exert their antimicrobial activity by a receptor-mediated mode-of-action^14–18^. In contrast, less potent eukaryotic AMPs appear in many cases to bind in a non-chiral manner to bacterial membrane lipids, and disrupt the membrane at sufficiently high peptide concentrations. For instance, the antimicrobial activity of the two-peptide bacteriocins plantaricin JK and lactococcin G depends on a putative amino acid transporter^17,18^ and a protein involved in cell wall synthesis^14^, respectively.

Previously, we^19–21^ and others^12,22–24^ have used circular dichroism (CD) spectroscopy and/or nuclear magnetic resonance (NMR) spectroscopy to characterize the structures of the peptides that constitute two-peptide bacteriocins. Neither of these class IIb bacteriocins were structured in water, but all became structured in membrane mimicking solutions. CD spectroscopy on plantaricin EF, plantaricin JK and lactococcin G have shown that when the peptides of two-peptide bacteriocins were mixed prior to the addition of liposomes they became more structured than the sum of the individual parts^23,24^. Similarly, CD studies have revealed that the plantaricin S peptides are partly α-helical in 50% TFE/water and exhibited an additional α-helical structuring of 5% when combined^12^. These results show that the peptides constituting a two-peptide bacteriocin can interact *in vitro* without the presence of a receptor^23,24^. Furthermore, in molecular dynamics (MD) simulations, a model of plantaricin EF inserted perpendicularly to the membrane bilayer was observed to have a higher degree of α-helical structuring than when the peptides were positioned on the surface of the membrane^25,26^. The receptor-mediated mode-of-action apparently involves so-called membrane catalysis^27,28^, in which the two-peptide bacteriocins are drawn to the membrane where they become structured, potentially interact and form a dimer that inserts in an orientation capable of interacting with a receptor.

**Figure 1:**
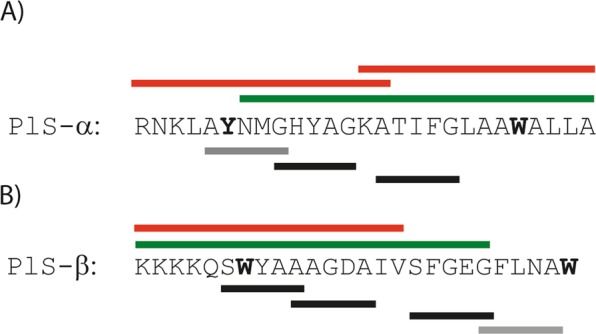
Sequences of the two peptides Pls-α (**A**) and Pls-β (**B**) investigated in this paper. Above the sequences, green bars illustrate sequence fragments that are active and red bars sequence fragments that are inactive according to Soliman *et al*.^12^. Below the sequences, the solid bars indicate the GxxxG and GxxxG-like motifs. The effect on activity by replacing the A, S or G residues in the black-barred A/S/GxxxA/S/G motifs with A, G or L and the bold W and Y residues with Y, W, L and R have been investigated in this paper.

GxxxG and GxxxG-like interaction motifs are generally believed to be important for helix-helix interactions in lipid membranes^29,30^. All class IIb bacteriocins characterized so far contain one or several GxxxG or GxxxG-like motifs where x is any amino acid flanked by small residues such as Gly, Ser and Ala^8^. Activity measurements and MD simulations indicate that these motifs are important for the antimicrobial activity of class IIb bacteriocins^19,25,31^. In plantaricin S there is one GxxxG motif in Pls-α (G_9_xxxG_13_) and several GxxxG-like motifs composed of Ala, Ser and/or Gly residues (Fig. 1). Soliman *et al*.^12^ performed antimicrobial activity assays of plantaricin S fragments and observed that the 21 C-terminal residues of Pls-α and 21 N-terminal residues of Pls-β were active^12^. Furthermore, CD spectroscopy indicated that the α-helix is in the C-terminal part of Pls-α. Based on these results it was suggested that the G_9_xxxG_13_ motif in Pls-α and the A_10_xxxA_14_ motif in Pls-β come close in space to form a transmembrane dimer complex. Interestingly, the N-terminal Pls-β fragment containing both the A_10_xxxA_14_ and S_17_xxxG_21_ motifs was active while a fragment lacking the latter motif was inactive, indicating that both these motifs may be important for activity (Fig. 1). Recently, a two-peptide bacteriocin called muricidin (Mur) was discovered in a *Lactobacillus murinus* strain^32^. Muricidin displays approximately 40–50% amino acid identity with plantaricin S, however, Mur-β lacks a AxxxA motif but contains a SxxxG motif that aligns with the S_17_xxxG_21_ motif in Pls-β. To our knowledge, the peptides of muricidin have neither been characterized, synthesized nor assayed to determining antimicrobial activity.

Here we determined the 3D structure of the two peptides constituting the two-peptide bacteriocin plantaricin S in DPC micelles applying NMR spectroscopy. To determine the importance of the GxxxG and GxxxG-like motifs in plantaricin S we replaced the small amino acids with other small (Ala, Gly) and larger (Leu) amino acids and measured the antimicrobial activity against indicator strains. Replacing an amino acid in any of the motifs in Fig. 1 involved in helix-helix interactions with e.g. larger side-chain residues is expected to severely affect the antimicrobial activity of the bacteriocin as this will disturb close and favorable inter-helical contacts due to steric interference^29^. On the other hand, substituting with other small residues is expected to be tolerated. In addition, we performed activity assays on combinations of the peptides constituting the bacteriocins plantaricin S and muricidin to determine whether the peptides could be interchanged and if the common motifs are important for the activity of plantaricin S. Aromatic residues such as Trp and Tyr may anchor transmembrane helices in the membrane-water interface^33,34^. Such amino acids were shown to be important for activity of lactococcin G^31^ and plantaricin EF^25^, as well as for the class IIa bacteriocin Sakacin P^35^. To gain insight into the positioning of the peptides in the membrane, antimicrobial activity of variants where the N- and C-terminal aromatic residues shown in Fig. 1 were single substituted with Trp, Tyr, Leu and Arg, has been determined.

## Results

### Circular dichroism

**Figure 2:**
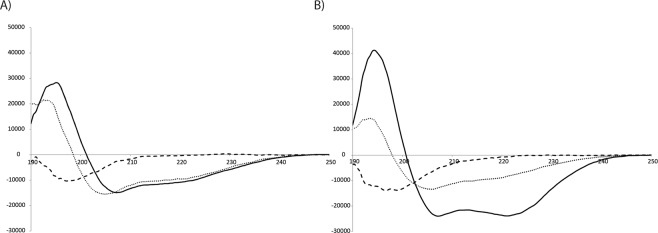
CD spectra of Pls-α in (**A**) and Pls-β in (**B**). The spectra in buffer solution, 50%/50% TFE/water, and 10 mM DPC are drawn with dashed, dotted and solid lines, respectively.

The CD spectra of Pls-α and Pls-β are shown in Fig. 2A,B, respectively. Both peptides are unstructured in buffer solution but become structured in TFE and DPC. While there is little difference in the degree of structuring of Pls-α in TFE and DPC, there is a huge difference for Pls-β as observed in Fig. 2B. Furthermore, the CD spectrum of Pls-α in TFE has a curve that seems to have a negative minimum shifted towards lower nm values than that observed in DPC (Fig. 2A). The CD results were best fitted, as evaluated based on RMSD and NMRSD values, using the routine Contin-ll in the CDpro program package^36^. The results are reported in supplementary section (Table S1). Due to the relatively drastic difference in the CD spectra in TFE and DPC, and the more bilayer-like properties of DPC, structure determination with NMR was done in DPC micelles. CD spectra of muricidin were recorded in similar environments (Supplementary Fig. S1). The results indicate that the lengths of the helices of the two bacteriocins are similar in TFE (Table S1). A difference, that probably is due to the difference in C-terminal residues was, however, observed between Mur-α and Pls-α in DPC.

### NMR structure determination

**Figure 3:**
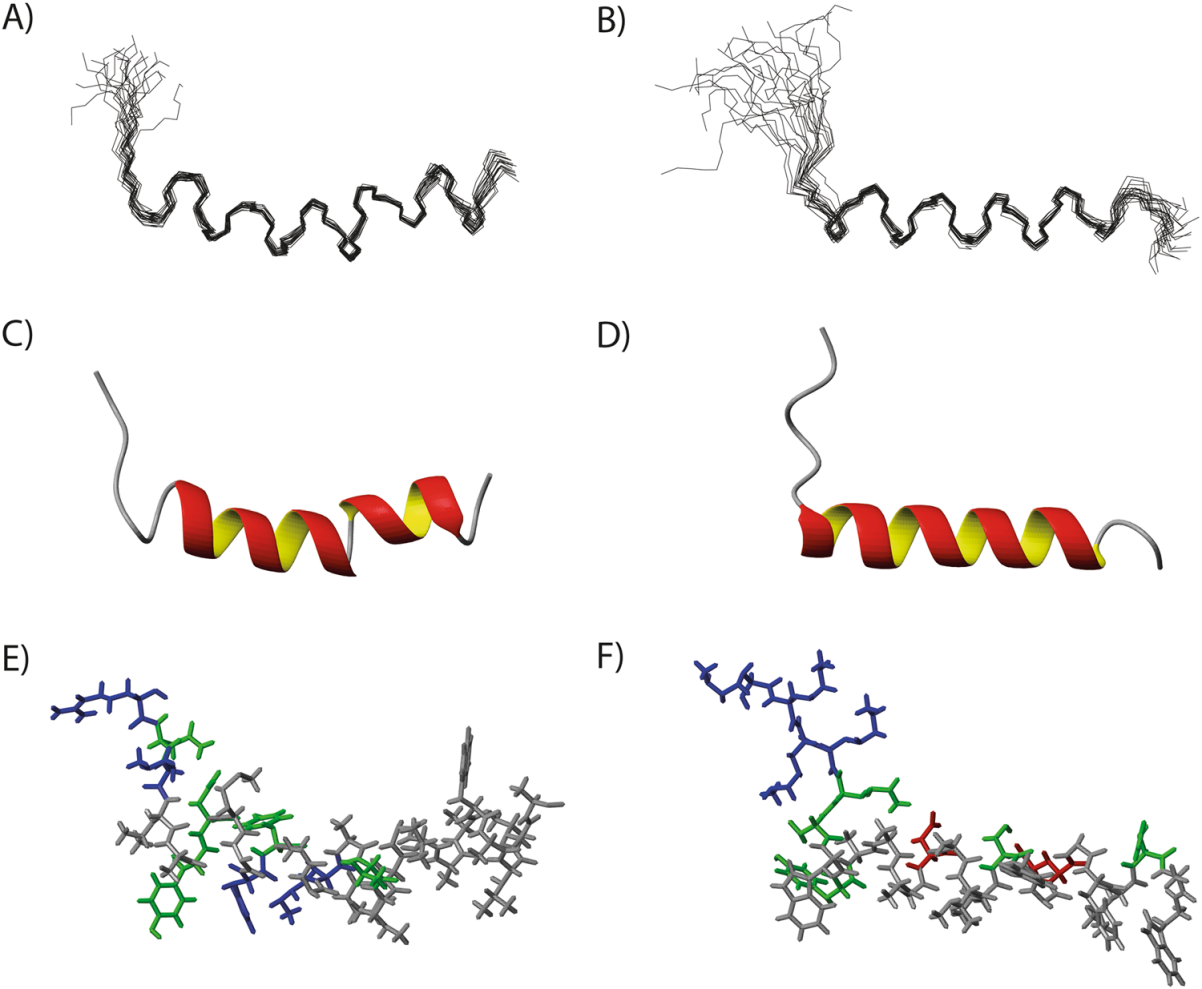
NMR structures of Pls-α (**A,C,E**) and Pls-β (**B,D,F**) in DPC micelles. (**A,B**) show the respective NMR structure ensembles of the 20 lowest energy structures. (**C,D**) show the cartoon representation of the structures. The atomic bonds with side chain residues are shown in (**E,F**). Positively charged, negatively charged, polar and non-polar residues are colored blue, red, green and light gray, respectively. All figures were generated by molmol^57^.

Chemical shifts were assigned using the 2D experiments described in methods and standard methods^37^. The assigned NOESY spectra are shown in supplementary section (Figs S2 and S3). The assigned chemical shifts have been uploaded to BMRB with the accession numbers 34278 and 34279. Chemical shift indexing^38^ indicates possible helical regions from and including residue 5 to 25 in Pls-α and from and including residue 7 to 22 in Pls-β (Supplementary Fig. S4A,B). Similar results were found by the application of TALOS+^39^ where the torsion angles indicated possible helical structuring from and including residue 6 to 26 in Pls-α and from and including residue 5 to 25 for Pls-β (Supplementary Fig. S4C,D). The sequence plot illustrating the short and medium range NOE correlations observed from the two peptides in the NOESY spectrum (Supplementary Fig. S4C,D) indicate a helical structure from residue 5 and out, with a potential helix break around residues 14 to 16, and from residue 7 to 21/22 for Pls-α and Pls-β, respectively. Structures calculated using restraints from NOESY and TALOS+ are shown in Fig. 3 while the structure restraints and structure ensemble characteristics are presented in supplementary section (Table S2).

The calculated and refined structures show that Pls-α contains a straight α-helix from residue 8 to 24 with a break around residue 18, while Pls-β has an α-helix from residue 7 to 23. The structures and the α-helical break observed in some of the determined structures of Pls-α reflects the limited number of medium restraints observed in Supplementary Fig. S4C. The structure ensembles (Fig. 3A,B), cartoon representation of the structures (Fig. 3C,D) and structures illustrating the charged residues (Fig. 3E,F) are shown in Fig. 3. Both Pls-α and Pls-β contain hydrophobic C-terminal residues, whereas the N-terminal region consists of Lys and polar residues. The N-terminal region of the α-helix of Pls-α is partly amphiphilic containing the positively charged or polar residues His10, Tyr11 and Lys14; however, the polar residue Thr16 is on the opposite side of the helix (Fig. 3E). All residues from 17 and out are hydrophobic, as is observable from the representation of Pls-α with side chains (Fig. 3E). The three C-terminal residues are unstructured, while the seven-residue long N-terminal region folds back along the helix of Pls-α. The C-terminal positioning of the helix is in agreement with the observation that there was a higher degree of helicity in TFE of Pls-α fragments containing 21 and 16 C-terminal residues than in the full length peptide^12^.

The α-helix of Pls-β is amphiphilic containing the two polar, negatively charged residues Asp13 and Glu20, with an unstructured, short C-terminal tail and an N-terminal region that is partly folding back on the helix (Fig. 3B,D,F). To our knowledge, no CD-data exist for fragments of Pls-β. However, the N-terminal helical region of LcnG-β, one of the two peptides constituting the two-peptide bacteriocin lactococcin G^12,21^, shares 38% sequence similarity with Pls-β, and a similar helix to the one observed here. The secondary structures of both Pls-α and Pls-β peptides are comparable to those found for other two-peptide bacteriocins^22^ and helicity is in reasonable agreement with the CD results. The structures of Pls-α and Pls-β have been published in the protein data bank (PDB) with the structural codes 6GNZ and 6GO0, respectively.

### Mutational effects on plantaricin S

Based on preliminary activity assays with plantaricin S against several bacterial strains, *Lactococcus lactis* IL1403 and *Lactobacillus sakei* NCFB 2714 were selected for further activity measurements of the mutated peptide variants (Supplementary Table S3).

The Gly, Ala and Ser residues of the putative helix-helix stabilizing motifs (Fig. 1) were single substituted with other small residues (Gly and Ala) or the large hydrophobic Leu residue. Furthermore, the terminal Trp and Tyr residues were substituted with Tyr, Trp, Leu and Arg to determine the membrane position of these residues. In total, 32 plantaricin S variants have been tested. Antimicrobial activity of peptide variants combined with the complementary wild type peptide was determined. Results of the GxxxG and GxxxG-like peptide variants in Table 1 are presented as minimum bacterial inhibitory concentration and relative minimum bacterial inhibitory concentration values with an ‘x-fold’ reduction (when x > 1) in activity of the peptide-combinations compared to the wild type minimum bacterial inhibitory concentration value. The antimicrobial activity measured was comparable between the two strains.

**Table 1:** Bacterial inhibitory concentration and relative bacterial inhibitory concentration values from activity measurements of GxxxG and GxxxG-like peptide variants of Pls-α and Pls-β together with the wild type complementary peptide.

Peptide combination^b^	Bacterial inhibitory concentration (nM)		Relative bacterial inhibitory concentration values^a^	
	*Lac. lactis* IL1403	*Lab. sakei* NCFB 2714	*Lac. lactis* IL1403	*Lab. sakei* NCFB 2714
α + β	0.6 ± 0.1	0.17 ± 0.04	1	1
α(G9A) + β	11.4 ± 0.9	3.7 ± 1.0	20	20
α(G9L) + β	>25	>25	>40	>150
α(G13A) + β	1.0 ± 0.1	0.36 ± 0.05	2	2
α(G13L) + β	>25	>25	>40	>150
α(A15G) + β	6.1 ± 1.5	1.25 ± 0.75	10	7
α(A15L) + β	0.7 ± 0.1	0.25 ± 0.04	1	1
α(G19A) + β	0.8 ± 0.2	0.22 ± 0.04	1	1
α(G19L) + β	4.3 ± 0.6	0.33 ± 0.02	7	2
β(S6G) + α	0.8 ± 0.1	0.23 ± 0.06	1	1
β(S6A) + α	0.6 ± 0.1	0.15 ± 0.05	1	1
β(S6L) + α	1.2 ± 0.1	0.29 ± 0.08	2	2
β(A10G) + α	1.7 ± 0.4	0.41 ± 0.12	2	2
β(A10L) + α	0.7 ± 0.1	0.26 ± 0.06	1	1
β(A14G) + α	3.1 ± 0.4	0.59 ± 0.09	5	3
β(A14L) + α	1.4 ± 0.1	0.36 ± 0.06	2	2
β(S17G) + α	5.4 ± 0.3	1.1 ± 0.35	9	6
β(S17A) + α	2.4 ± 0.2	0.51 ± 0.19	4	3
β(S17L) + α	10 ± 1	1.3 ± 0.9	17	8
β(G21A) + α	4.1 ± 0.3	1.0 ± 0.47	7	6
β(G21L) + α	>25	>25	>40	>150

^a^The relative bacterial inhibitory concentration is defined as a fold increase or decrease in activity compared to the wild type combination.

^b^The peptides were added in equimolar amounts. α and β are Pls-α and Pls-β, respectively. Single amino acid substitutions are indicated in parentheses.

Replacing the Gly9 in Pls-α with an Ala residue reduced the activity 20-fold whereas this substitution was tolerated in position 13. However, replacing Gly residues (at position 9 and 13), with a Leu residue was highly detrimental, suggesting that the G_9_xxxG_13_ motif in Pls-α is important for activity of the bacteriocin. On the contrary, the A_15_xxxG_19_ motif is not important in helix-helix interactions as substituting with a Leu residue at these positions only slightly reduced the activity (1- to 5-fold). Substituting Ala15 with a Gly residue was somewhat detrimental, perhaps due to the more flexible Gly residue, which may destabilize the α-helical structure. For the Pls-β peptide, neither the S_6_xxxA_10_ nor the A_10_xxxA_14_ motifs had strong impact on the activity when substituted with Gly, Ala or Leu residues. Substituting Ser17 with a Leu residue in the S_17_xxxG_21_ motif reduced the activity the most at this position compared to a Gly and an Ala residue, which reduced the activity 5- to 10-fold. Similar results were observed for Gly21 where replacing Gly with a Leu residue were detrimental on activity. These results indicate that the G_9_xxxG_13_ motif in Pls-α and the S_17_xxxG_21_ motif in Pls-β are important for bacteriocin activity.

The effect of single amino acid substitutions of the terminal Trp and Tyr residues of plantaricin S (Supplementary Table S4) showed that replacing Pls-α Y6 and Pls-β W26 with Arg were detrimental, while tolerated relatively well in Pls-α W23 and Pls-β W7. Replacement with Leu or Trp/Tyr residues was tolerated in all four positions.

### Activity of plantaricin S and muricidin peptide combinations

To investigate if the recently discovered bacteriocin muricidin^32^ possesses antimicrobial activity, we performed activity assays of the individual peptides and combinations of muricidin and plantaricin S peptides (Table 2). The minimum bacterial inhibitory concentration of muricidin was 2 to 3 times higher than that observed for plantaricin S against the two indicator strains tested. No or weak antimicrobial activity was observed for Mur-α and Mur-β individually. The fact that Mur-α has no individual activity within the concentrations tested, whereas the activity increases approximately 5000 times when Mur-α and Mur-β are combined as compared to Mur-β alone, clearly shows that muricidin is a two-peptide bacteriocin as predicted by Collins *et al*.^32^. Due to sequence similarities with plantaricin S, the activity of the combination of individual peptides of plantaricin S and muricidin was tested. Interestingly, the combinations Pls-α with Mur-β and Pls-β with Mur-α displayed high antimicrobial activity, only reduced by 3 to 5 times compared to plantaricin S.

**Table 2:** Bacterial inhibitory concentration and relative bacterial inhibitory concentration values from activity measurements of wild type combinations of Pls-α, Pls-β, Mur-α and Mur-β.

Peptide combination^b^	Bacterial inhibitory concentration (nM)		Relative bacterial inhibitory concentration values^a^	
	*Lac. lactis* IL1403	*Lab. sakei* NCFB 2714	*Lac. lactis* IL1403	*Lab. sakei* NCFB 2714
Pls-α	>1000	91 ± 5	>1500	650
Pls-β	>1000	>1000	>1500	>7000
Mur-α	>1000	>1000	>1500	>7000
Mur-β	>1000	670 ± 120	>1500	5000
Pls-α + Pls-β	0.7 ± 0.2	0.14 ± 0.02	1	1
Mur-α + Mur-β	2.1 ± 0.4	0.23 ± 0.06	3	2
Pls-α + Mur-α	>1000	26 ± 5	>1400	200
Pls-α + Mur-β	2.2 ± 0.3	0.63 ± 0.01	3	5
Pls-β + Mur**-**α	3.2 ± 0.7	0.63 ± 0.01	5	5
Pls-β + Mur-β	>1000	270 ± 120	>1400	2000

^a^The relative bacterial inhibitory concentration is defined as a fold increase or decrease in activity compared to the wild type combination of Pls-α and Pls-β.

## Discussion

In this study, we present the NMR structures of two peptides Pls-α and Pls-β, that constitute the bacteriocin plantaricin S, in DPC micelles and the CD structures of Pls-α, Pls-β, Mur-α and Pls-β in water, TFE and DPC micellar solutions. The peptides are unstructured in water and become α-helical upon interaction with membrane-mimicking environments^12,19–24^. The α-helical structures determined by NMR of the two plantaricin S peptides are in agreement with the CD results, and the structures are similar to those observed for other two-peptide bacteriocins and proposed by Soliman *et al*.^12^. Previous predictions, however, indicated that the helices were from residues 15 to 25 and 4 to 15^12^. The structures determined here show that the peptides form linear helices from residue 8–24 with a break around residue 18 and 7–23 for Pls-α and Pls-β, respectively. Thus, the helices in the determined structures are both longer and positioned more centrally in the peptides than previously suggested.

### GxxxG and GxxxG-like motifs important for plantaricin S activity

Several activity studies indicate that the GxxxG and GxxxG-like motifs, identified in all characterized class IIb two-peptide bacteriocins so far, are important for the bacteriocin activity^19,25,31^ in allowing close contact and stabilizing interactions between helices.

A sequence alignment of Pls-α and Pls-β with two-peptide bacteriocins with, to our knowledge, most similar sequences are shown in Table 3. The sequence alignment of Pls-α, Mur-α and plantaricin E (PlnE)^40^ illustrates that the G_9_xxxG_13_ motif in Pls-α aligns perfectly with G_9_xxxG_13_ in Mur-α and with the G_5_xxxG_9_ motif found to be important for PlnE^25^. The alignment, furthermore, illustrates a higher degree of sequence similarity between Pls-α and Mur-α in the helix portion of Pls-α, residues 8 to 15, than in the more flexible N- and C-terminal parts. In agreement with the findings of Soliman *et al*.^12^, the sequence alignments and the activity measurements indicate that the G_9_xxxG_13_ motif in Pls-α is highly important for activity.

**Table 3:** Sequence alignment of individual peptides of two-peptide bacteriocins illustrating identical or similar residues.

Peptide	Amino acid sequence^a^
	
Pls-α	
Mur-α	
Plantaricin E^b^^40^	
	
Pls-β	
Mur-β	
Lactococcin G-β^58^	

^a^Hyphens indicate amino acids that are not present in these peptides, bold letters indicate motifs identified to be important for bacteriocin activity and underlined letters indicate conserved or similar residues in plantaricin S that correspond to the important motifs identified in the other peptides. Bold/italic and italic boxes illustrate amino acids that are identical or similar, respectively, to the individual peptides of plantaricin S.

^b^There are at least two ways that plantaricin E^40^ can be aligned with Pls-α.

^c^Numbering with regards to the sequence of plantaricin S.

Soliman *et al*.^12^ showed that a 21-residue long N-terminal fragment that includes the S_17_xxxG_21_ motif had activity similar to the wild type peptide when combined with Pls-β, however, a fragment that only included the A_10_xxxA_14_ motif in Pls-β was inactive. This is in agreement with the finding that single amino acid changes were less tolerated in the S_17_xxxG_21_ motif than in other investigated motifs in Pls-β. Table 3 shows that the S_17_xxxG_21_ motif in Pls-β, the S_14_xxxG_18_ motif in Mur-β and the G_18_xxxG_22_ motif that previously has been shown to be important for lactococcin G-β (LcnG-β) activity^31^ align, while neither the G_18_xxxG_22_ motif in LcnG-β nor other N-terminal GxxxG and GxxxG-like motifs in Mur-β align with A_10_xxxA_14_ in Pls-β. Furthermore, only minor changes in activity were observed when single amino acid replacements were performed in the previously predicted interaction motif A_10_xxxA_14_ in Pls-β (Table 1). The observation that S_17_xxxG_21_ motif in Pls-β is important for antimicrobial activity is not unique in that a SxxxG motif was also observed to be important in PlnF for its interactions with the complementary peptide PlnE in the bacteriocin plantaricin EF^25^.

### Combinations of peptides from different bacteriocins are active

Activity assays on muricidin and the individual peptides that constitute this bacteriocin are reported here for the first time. Due to the large sequence similarities between the peptides of plantaricin S and muricidin, the activities of bacteriocin combinations were assayed to determine whether or not combinations of the peptides from different bacteriocins are active (Table 2). The near plantaricin S activity of combinations of Pls-α and Mur-β and Pls-β and Mur-α (Table 2) indicate that motifs the two peptides have in common (Table 3) are important for their activity.

The nM minimum bacterial inhibitory concentration values observed for both plantaricin S, muricidin and peptide combinations further indicate that the bacteriocins act through a common receptor and not directly on the membrane, similarly to what has been observed for other one- and two-peptide bacteriocins^14,15,17,18^. No such receptor is, however, described for either of the bacteriocins in the literature to our knowledge. The highly similar activities further indicate that the identical residues, which are mainly located in the α-helices of the peptides, are important for receptor interaction and that several residue changes may be accepted in the bacteriocin as long as these are not directly or indirectly affecting receptor interaction.

### Dimer model of plantaricin S in bilayer

In order for the two-peptide bacteriocin to exert an antimicrobial effect, the complementary peptides need to obtain the correct conformation to act on a receptor. The degree of structuring of most two-peptide bacteriocins, as was the case for Pls-α and Pls-β^12^, increases when combined *in vitro* compared to the sum of the parts^23,24^. We believe that this increased structuring is important for the receptor interaction, and that the structuring appear by the following mechanism; First the peptides of plantaricin S become structured in the membrane interface, then they interact and become one active bacteriocin dimer that inserts into the membrane, resulting in increased structuring of the bacteriocin. Similar mechanisms for the formation of bacteriocin dimers have previously been proposed for plantaricin S^12^ and other two-peptide bacteriocins^19,25,31^. This membrane insertion is believed to be a prerequisite for the interaction of bacteriocins with a membrane-bound receptor^27,28^. It should be noted that at least in one two-peptide bacteriocin, carnobacteriocin XY, no *in vitro* interaction between the peptides has been observed^22^.

**Figure 4:**
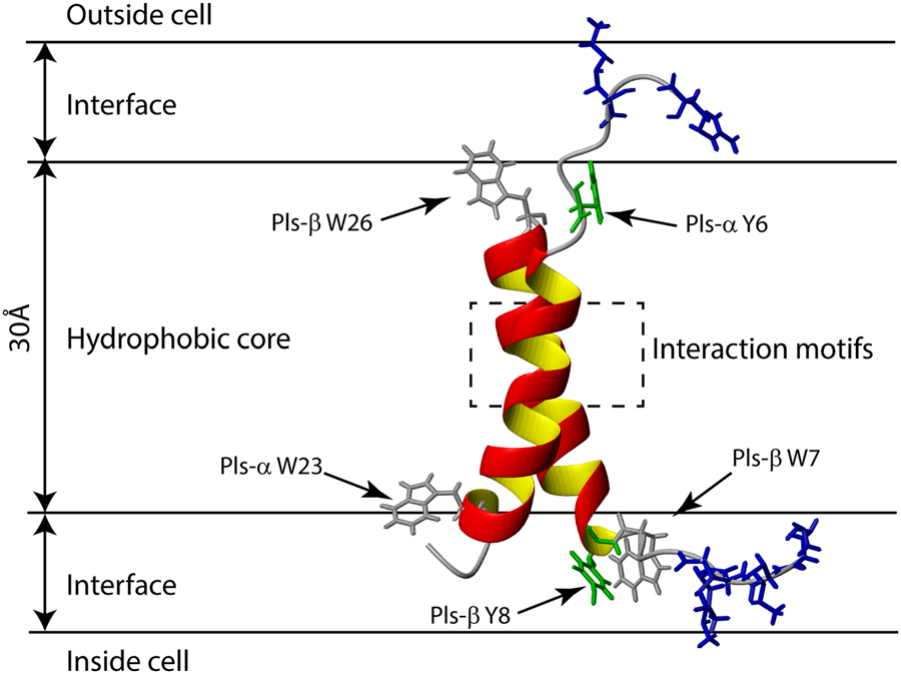
Proposed antiparallel model of the bacteriocin plantaricin S inserted into a membrane bilayer. Structures are drawn in cartoon format, Trp and Tyr residues are shown with side chains colored in light gray and green, respectively, while side chains of N-terminal positively charged residues are colored in blue. The proposed interaction motifs are inside the stapled box.

Plantaricin S can form a parallel or anti-parallel transmembrane dimer complex when the G_9_xxxG_13_ motif in Pls-α is in close contact with the S_17_xxxG_21_ motif in Pls-β. If the G_9_xxxG_13_ motif in Pls-α is close to the S_17_xxxG_21_ motif in Pls-β in a parallel orientation, there will be little helix-helix overlap as the two motifs are in the N- and C-terminal ends of the respective helices. However, there is greater overlap between the helical regions of the two peptides in an antiparallel orientation. Activity of the variants where aromatic amino acids were replaced (Supplementary Table S4) are in overall agreement with the proposed model positioning the terminal aromatic residues Tyr and Trp in the membrane-water interface as illustrated in Fig. 4. The detrimental effect of replacing Pls-α Y6 and Pls-β W26 with Arg indicates that these residues are either positioned deeper into the bilayer than Pls-α W23 and Pls-β W7 or that the introduction of a positive charge affects the interaction with a receptor. Interestingly, neither of the active fragments observed by Soliman *et al*.^12^ contain these aromatic residues, indicating that specific receptor interactions may be of importance. Replacing Ala15 and Gly19 in Pls-α and Ala10 and Ala14 in Pls-β with a Leu residue were well tolerated, thus these Ala residues may be positioned in the hydrophobic core of the membrane protected from water, in agreement with the proposed model (Fig. 4). Furthermore, the substitutions of Ser6 in Pls-β (Table 1) resulted in wild type activity, indicating that this polar residue is close to the membrane-water interface which also agrees with the proposed model. According to the positive inside rule^41^, the positively charged N-terminal of Pls-β is expected to be positioned on the cytosolic side of the membrane as illustrated in the model (Fig. 4). Previous MD simulations on plantaricin S, where the G_9_xxxG_13_ motif in Pls-α was paired with the A_10_xxxA_14_ motif in Pls-β, shows that this antiparallel model was able to partly insert into the upper membrane leaflet while the parallel model was not^12^. Unfortunately, no trajectories of the simulation were shown in the paper, making investigation into the model impossible. The relatively short length of the simulation (400 ns) compared to the several seconds full insertion may take^42,43^, may explain why the antiparallel dimer suggested here was not formed.

## Materials and Methods

### Synthetic peptides

The wild type Pls-α, Pls-β, Mur-α and Mur-β peptides and single amino acid-mutated variants were purchased from GenScript. All peptides used in CD spectroscopy and NMR spectroscopy were ordered at a purity of >98%, while the peptides only used for activity measurement were ordered with a purity of >80%. Peptides used in activity measurements were dissolved in 40% 2-propanol upon arrival and stored at −20 °C, while the peptides used for structure investigation were dissolved on the day of use. The peptide concentrations were determined based on the molar extinction coefficients of the amino acids Tyr (ε_280_ = 1200 M^−1^ cm^−1^) and Trp (ε_280_ = 5560 M^−1^ cm^−1^) and absorption measured spectrophotometrically at 280 nm.

### CD spectroscopy

CD spectra were recorded using a Jasco J-810 spectropolarimeter (Jasco International Co) calibrated with D-camphor-10-sulfonate (Icatayama Chemical). All measurements were done using a quartz cuvette (Starna) with 0.1 cm path length. Samples were scanned five times with a scanning rate of 50 nm/min with a bandwidth of 0.5 nm and a response time of 1 s over the wavelength range 190–260 nm (TFE and DPC samples). Spectra were recorded at 0, 30, 40 and 50% trifluoroethanol (TFE; Sigma-Aldrich) and in 8, 10 and 12 mM dodecylphosphocholine (DPC; Avanti Polar lipids) concentrations at 25 °C. The approximate α-helical content of the protein was determined using CDpro^36^.

### NMR sample preparation, NMR spectroscopy and structure calculations

The experiments were run on a sample containing 1 mM of the peptide, 100 mM D38-DPC (Sigma-Aldrich), 5% D_2_O (Sigma-Aldrich), Milli-Q water and 0.2 mM of 4,4-dimethyl-4-silapentane-1-sulfonic acid (DSS; Larodan).

2D NOESY^44^, 2D TOCSY^45^, 2D DQCOSY, ^15^N-HSQC^46^ and ^13^C-HSQC^47^ NMR spectra were recorded. The data were acquired on a 600 MHz Bruker Avance II spectrometer with four channels and a 5 mm TCI cryoprobe (Bruker Biospin). NOESY and TOCSY spectra with mixing times of 120–300 ms and 60–80 ms, respectively, were used and the experiments were run at 298.15 K. Spectra were processed using the Topspin program (Bruker Biospin). DSS was used as a chemical shift standard, and ^15^N and ^13^C data were referenced using frequency ratios^48^.

Visualization and assignment of the spectra were performed using the computer program CARA^49^ while SPARKY^50^ was used for integration of NOESY peaks. The spectra were assigned with standard methods^37^. Dihedral angle restraints were obtained from the chemical shift values using the program TALOS+^39^. Nuclear Overhauser effect (NOE) distance restraints were calculated from the peak volumes in the NOESY spectra with a NOESY mixing time of 120 ms. Cyana 2.1^51,52^ was used for NOE assignment and restraint calculations. Structure calculations were done using CNS^53,54^ and refinement in DMSO was done applying all restraints and RECOORD^55^. 100 structures were calculate and refined, the 20 structures with the lowest energy were analyzed further. The number of applied restraints and structure statistics are presented in Supplementary Table 1.

### Bacterial strains, growth conditions and activity measurements

Indicator strains were obtained from Laboratory of Microbial Gene Technology, Department of Chemistry Biotechnology and Food Science, Norwegian University of Life Science, Norway. All strains were grown at 30 °C without agitation in de Man-Rogosa-Sharpe (MRS) medium (Oxoid). The indicator strains *Lac. lactis* IL1403 and *Lab. sakei* NCFB 2714 were selected for mutational analysis based on sensitivity towards plantaricin S (Supplementary Table S3).

Antimicrobial activity of wild type and variants of Pls-α and Pls-β were analyzed in a microtiter plate assay system, essentially as described by Nissen-Meyer *et al*.^56^. In brief, each well of the microtiter plate contained MRS medium to a final volume of 200 μl, combinations of wild type and mutated variants of Pls-α and Pls-β (in 1:1 ratio), and indicator strain. The individual peptides were added at a concentration of 25 nM in the first well, with a two-fold dilution factor going from one well to the next. The muricidin peptides were added at a concentration of 25 nM in the first well, while the peptides of both muricidin and plantaricin S were added at 1000 nM concentrations for determining individual activity. Peptide combinations with decreased antimicrobial activity compared to the wild type combination were added at individual concentrations of 100 nM in the first well. Freshly made stationary phase cultures of indicator strains were diluted 1:50 and the microtiter plates were incubated for 5 hours at 30 °C. The growth of the indicator cells was measured spectrophotometrically at 600 nm by use of a Sunrise^™^ Remote microplate reader (Tecan).

The bacterial inhibitory concentration was defined as the total amount of peptides added, at a 1:1 ratio, which inhibited the growth of the indicator strain by 50%. The relative bacterial inhibitory concentration value was quantitated in terms of fold increase or decrease in antimicrobial activity compared to the wild type combination.

## Supplementary information

Supplementary
