# Supplementary material for: NMR structures and mutational analysis of the two peptides constituting the bacteriocin plantaricin S
Source: Sci Rep. 2019 Feb 20;9:2333. doi: 10.1038/s41598-019-38518-6 (PMC6382864; doi:10.1038/s41598-019-38518-6)
Supplement: Supplementary file 1 — Supplementary [file 41598_2019_38518_MOESM1_ESM.docx]

# Supplementary section to the paper entitled: NMR structures and mutational analysis of the two peptides constituting the bacteriocin plantaricin S

Bie Ekblad and Per Eugen Kristiansen*

Department of Biosciences, University of Oslo, PO Box 1066, Blindern, NO-0316 Oslo, Norway.

*****Corresponding author: Per Eugen Kristiansen; p.e.kristiansen@ibv.uio.no

**Abbreviations used in the supplementary material.**

**CD** circular dichroism

**DPC** dodecyl phosphocholine

**NMR** nuclear magnetic resonance

**Pls-α** plantaricin S α

**Pls-β** plantaricin S β

**TFE** 2,2,2-Trifluoroethanol

**Supplementary figures:**

**

**

**Figure S1** CD spectra of Mur-α in **A)** and Mur-β in **B)**. The spectra in buffer solution, 50%/50% TFE/water, and 10 mM DPC are drawn with dashed, dotted and solid lines, respectively.





**Figure S2.** The NOESY spectrum of Pls-α in 100 mM DPC micellar solution with selected assignments





**Figure S3.** The NOESY spectrum of Pls-β in 100 mM DPC micellar solution with selected assignments





**Figure S4.** Chemical shift indexes of Pls-α **A)** and Pls-β **B).** Sequence plots of the sequential and medium range NOE restraints used in structure calculation of Pls-α **C)** and Pls-β **D)**. The stars indicate residues where Talos predicted torsion angles in the α-helical region of the Ramachandran plot. The horizontal lines indicate NOE cross peak between the protons in different amino acids. Line thickness is related to NOE cross peak intensity.

**Supplementary tables:**

**Table S1.** The degree of helicity determined for plantaricin S and muricidin from CD spectra by the procedure Contin-ll in CDpro^1^

|  | **Pls-α** | | | **Pls-β** | | | **Mur-α** | | **Mur-β** | |
| --- | --- | --- | --- | --- | --- | --- | --- | --- | --- | --- |
|  | | **%** | Number of residues^a)^ | **%** | Number of residues^a)^ | **%** | | Number of residues^a)^ | **%** | Number of residues^a)^ |
| **50% TFE** | | 48.9 ± 0.8 | 13-14 | 42.6 ± 1.1 | 11 | 35.0 ± 1.1 | | 10 | 57.3 ± 1.8 | 13 |
| **10mM DPC** | | 49.0 ± 1.3 | 13-14 | 67.3 ± 1.4 | 17-18 | 24.1 ± 1.3 | | 7 | 70.5 ± 0.7 | 16 |

^a)^ Calculated from the % helicity and the length of the peptides

**Table S2.** Restraints used in structure calculation, energies, torsion angle- and RMSD characteristics of the final NMR ensembles of Pls-α and Pls-β.

|  |  | **Pls-α** | **Pls-β** |
| --- | --- | --- | --- |
| **Conformationally restricting restraints** |  |  |  |
| NOE | Intra | 108 | 93 |
|  | Sequential | 147 | 129 |
|  | Medium range | 176 | 125 |
|  | Total | 432 | 347 |
| Dihedral |  | 44 | 40 |
| Energies ^a)^ | CNS energy | -6535 ± 45 | -7036 ± 51 |
|  |  |  |  |
| **Residual restraint violations ^b)^** |  |  |  |
| Average number of distance violations per structure | 0.1-0.2Å | 1.3 | 2.4 |
| Average number of distance violations per structure | >0.2Å | 0 | 0.25 |
| Largest distance violation |  | 0.18 | 0.36 |
| Average number of dihedral angle violations | > 5^0^ | 0 | 0 |
| Largest dihedral angle violation |  | 3.8 | 3.4 |
|  |  |  |  |
| **Model quality** |  |  |  |
| Dihedral ^c) d)^ | Most favored | 98.2 | 96.3 |
|  | Additional allowed | 1.2 | 3.5 |
|  | Generously allowed | 0.4 | 0.2 |
| RMSD | All residues^e)^ | 0.92 ± 0.30  (1.93 ± 0.50) | 1.89± 0.63  (3.05 ± 0.67) |
|  | Residue 6-24^e)^ | 0.42 ± 0.12  (0.96 ± 0.19) | 0.59 ± 0.25  (1.09 ± 0.28) |
|  | RMS deviation bond lengths (Å) ^c)^ | 0.014 | 0.014 |
|  | RMS deviation angle (^0^)^c)^ | 1.1 | 1.1 |
| Model quality scores | Molprobity clash score ^c) f)^ | 18.18 (-1.59) | 7.41 (0.25) |
|  | Procheck (all) ^c) f)^ | -0.16 (-0.95) | 0.06 (0.35) |
|  | Procheck (phi-psi) ^c) f)^ | -0.18 (-0.39) | 0.06 (0.55) |

1. CNS energy values ^2,3^ after refinement in DMSO using all restraints and the RECOORD script.^4^
2. Determined with PDBstat^5^
3. Determined with PSVS^6^
4. Determined with Molprobity^7^
5. RMSD values are for the backbone atoms (N, CA, CB, C and O) calculated using MolMol ^8^ and values in parenthesis is for all heavy atoms.
6. Values Raw scores with the Z-score are in parenthesis.

**Table S3** Plantaricin S bacterial inhibitory concentration values for tested strains.

| Strain | LMGT# | Bacterial inhibitory concentrations (nM) |
| --- | --- | --- |
| *Weissella viridescens* NCFB 1655 | 2314 | 1,2/1 |
| *Lactabacillus* *curvatus* 89 | 2355 | 0,5/0,3 |
| *Lactobacillus plantarum* 965 | 2003 | 5,3/3,5/3,4/4,3 |
| *Lactobacillus sakei NCFB 2714* | 2313 | 0,2/0,2 |
| *Pediococcus pentosaceus* NCFB 990 | 2315 | 12/23,4 |
| *Pediococcus acidilatici* NCFB 1859 | 2316 | 61/37 |
| *Enterococcus faecalis* NCFB 581 | 2602 | 5,3/4,8 |
| *Enterococcus faecalis* | 2333 | 16/8,8 |
| *Lactococcus lactis* IL1403 | 1403 | 0,5/0,6/0,3 |
| *Lactococcus lactis MG1363* | 1363 | 6,9/4 |

Bacterial inhibitory concentration values for individual bacterial growths tested are separated by a bar. Bacterial strains were obtained from Laboratory of Microbial Gene Technology, Department of Chemistry Biotechnology and Food Science, Norwegian University of Life Science, Norway. LMGT# refers to the microbial library code.

**Table S4** The effect of single amino acid replacement substitutions of selected Trp and Tyr residues

|  | **Bacterial inhibitory concentration (nM)** | | **Relative bacterial inhibitory concentration values^a)^** | |
| --- | --- | --- | --- | --- |
| **Peptide combination** ^b)^ | ***Lac. lactis* IL1403** | ***Lab. sakei* NCFB 2714** | ***Lac. lactis* IL1403** | ***Lab. sakei* NCFB 2714** |
| α + β | 0.7 ± 0.2 | 0.14 ± 0.02 | 1 | 1 |
| α(Y6L) + β | 2.2 ± 0.3 | 0.67 ±0.02 | 3 | 5 |
| α(Y6R) + β | 79 ± 4 | 16 ± 2 | 110 | 110 |
| α(Y6W) + β | 1.2 ± 0.1 | 0.27 ±0.03 | 2 | 2 |
| α(W23L) + β | 0.8 ± 0.3 | 0.13 ± 0.03 | 1 | 1 |
| α(W23R) + β | 2 ± 0.5 | 0.96 ±0.17 | 3 | 7 |
| α(W23Y) + β | 0.4 ± 0.1 | 0.12 ±0.01 | 0.6 | 1 |
| α + β(W7L) | 1.9 ± 0.5 | 0.43 ± 0.05 | 3 | 3 |
| α + β(W7R) | 2.4 ± 0.5 | 0.55 ±0.04 | 3 | 4 |
| α + β(W7Y) | 2.3 ± 0.6 | 0.59 ± 0.07 | 3 | 4 |
| α + β(W26L) | 1.8 ± 0.5 | 0.35 ± 0.04 | 3 | 3 |
| α + β(W26R) | 17 ± 4 | 3.5 ±1.2 | 24 | 25 |
| α + β(W26Y) | 2 ± 0.5 | 0.47 ±0.11 | 3 | 3 |

^a)^ The relative bacterial inhibitory concentration is defined as a fold increase or decrease in activity compared to the wild type combination.

^b)^ The peptides were added in equimolar amounts. α and β are plantaricin S-α and plantaricin S-β, respectively. Single amino acid substitutions are indicated in parentheses.

**References:**

1 Sreerama, N. & Woody, R. W. Analysis of protein CD spectra: Comparison of CONTIN, SELCON3, and CDSSTR methods in CDPro software. *Biophys. J.* **78**, 334A-334A (2000).

2 Brunger, A. T. *et al.* Crystallography & NMR system: A new software suite for macromolecular structure determination. *Acta crystallographica. Section D, Biological crystallography* **54**, 905-921 (1998).

3 Brunger, A. T. Version 1.2 of the Crystallography and NMR system. *Nature Protocols* **2**, 2728-2733, doi:10.1038/nprot.2007.406 (2007).

4 Nederveen, A. J. *et al.* RECOORD: a recalculated coordinate database of 500+ proteins from the PDB using restraints from the BioMagResBank. *Proteins* **59**, 662-672, doi:10.1002/prot.20408 (2005).

5 Tejero, R., Snyder, D., Mao, B., Aramini, J. M. & Montelione, G. T. PDBStat: a universal restraint converter and restraint analysis software package for protein NMR. *J Biomol Nmr* **56**, 337-351, doi:10.1007/s10858-013-9753-7 (2013).

6 Bhattacharya, A., Tejero, R. & Montelione, G. T. Evaluating protein structures determined by structural genomics consortia. *Proteins* **66**, 778-795, doi:10.1002/prot.21165 (2007).

7 Chen, V. B. *et al.* MolProbity: all-atom structure validation for macromolecular crystallography. *Acta crystallographica. Section D, Biological crystallography* **66**, 12-21, doi:10.1107/s0907444909042073 (2010).

8 Koradi, R., Billeter, M. & Wüthrich, K. MOLMOL: A program for display and analysis of macromolecular structures. *J. Mol. Graph.* **14**, 51-55, doi:<http://dx.doi.org/10.1016/0263-7855(96)00009-4> (1996).
